# Brazilian immunology in Caxambu: beyond vaccination, a tribute to the pioneers of basic research in Chagas disease

**DOI:** 10.1590/0074-02760210314chgsb

**Published:** 2022-05-09

**Authors:** Julio Scharfstein

**Affiliations:** 1 Universidade Federal do Rio de Janeiro, Instituto de Biofísica Carlos Chagas Filho, Laboratório de Imunologia Molecular e Celular

In response to the editors’ question “Why don’t we develop a vaccine against Chagas disease”, Camargo, Morel, Gazzinelli, and Precioso provided a broad overview of the challenges that the Brazilian scientific community has confronted in its mission to prevent heart and digestive system pathology through vaccination against *Trypanosoma cruzi*. In his historical introduction, Erney Camargo, the scientist leader that transformed the National Research Council (CNPq) into a dynamic vector of scientific growth during his presidency in the 90’s, started his section reminding us that PIDE, a funding program that supported research on endemic diseases in the mid 70’s, was instrumental for the development of basic research in Chagas disease. Led by an iconic figure, Zigman Brener, the pioneers of research in our field organised (in 1994 onwards) the Annual Meetings of Basic Research on Chagas Disease, Caxambu. If the Hotel Gloria stays in our memory as a “temple” of *T. cruzi* research, Zigman Brenner and Zilton Andrade provided us with a “Bible”: the book “*Trypanosoma cruzi* and Chagas Disease”, first edited in 1979, included 16 chapters written by 16 Brazilian authors. Bound in a brown cover, that book was a fundamental source of knowledge for the younger generation of scientists and students that every November, stormed Caxambu to present the studies that gradually sculptured the landscape of Brazilian science in general, and immunology, in particular. In the early 80’s, research focused on vaccine development expanded vigorously under the impact of revolutionary discoveries in basic immunology (monoclonal antibodies, T cell recognition of MHC/peptide structure-function), biochemistry, and molecular and cellular biology (gene cloning and screening of expression libraries). In the early 80’s, Nobuko Yoshida, working as a postdoc at NYU under Ruth and Victor Nussenzweig leadership, made a breakthrough using monoclonal antibodies to characterise the immunodominant antigen (CS protein) of malaria sporozoites.^1^ ^)^ That project, which rapidly led to gene cloning and expression of recombinant CS, became a paradigm for vaccine research. By the same time, Nadia Nogueira, a Brazilian scientist that was actively studying cellular immunity in *T. cruzi* infection at Rockefeller University made a twist in her research by focusing on humoral immunity: for the first time, radio-iodinated surface glycoproteins expressed by trypomastigotes (mammalian-dwelling stages) were immunoprecipitated and characterised.^2^ During that period, the genetic and phenotypic diversity of the *T. cruzi* species was unveiled by molecular epidemiology experts in UK, Brazil and elsewhere. Concurrent with these advances, biochemists and cellular biologists pooled efforts to investigate the role of surface adhesion molecules in host-cell invasion by trypomastigotes. Using monoclonal antibodies directed against members of the *trans*-sialidase (TS) family, Sergio Schenkman (EPM/UNIFESP) and Julia M Alves Filho and Walter Colli (USP) provided independent evidence that host-cell invasion was at least partially dependent on the adhesive interactions mediated by TS/Tc85.^3^ ^,^ ^4^ The vision that TS should be incorporated in vaccine constructs was strengthened by studies performed by Carlos Frasch and co-workers in Argentina, showing that the SAPA domain of TS (composed of tandem-repeat epitopes) was target of protective antibodies in *T. cruzi*-infected mice. Since trypomastigotes express active TS at late stages of intracellular morphogenesis, there was a risk that the display of MHC-I/peptides at the surface of infected cells would be delayed, hence limiting target recognition and killing by cytotoxic CD8+ T cells. Playing safe, Maurício M Rodrigues, Gazzinelli and co-workers overcame this limitation by associating the TS gene to ASP-2,^5^ ^)^ a member of the active family of the TS family that is expressed (earlier) by intracellular amastigotes. Although these vaccine constructs proved highly efficacious in mice challenged by *T. cruzi* strains, few of us would doubt that vaccine efficacy in endemic areas might be limited due to the genetic diversity of the *T. cruzi* species. This having said, vaccines based on TS/ASP-2 constructs might be further improved by incorporating gene sequences (T-cell epitopes) from highly conserved *T. cruzi* antigens, such as Tc24, ribosomal proteins, cruzipain, or other conserved ORF sequences of *T. cruzi* genome. For example, chagasin, a tight-binding inhibitor of cruzipain^6^ which is evolutionary conserved in several parasitic protozoa comes to mind because its primary structure is unrelated to the functionally related cystatins of mammalian origin.

As acknowledged by Gazzinelli, the use of conventional mouse strains in preclinical trials of vaccine against *T. cruzi* has two major shortcomings. First, it is well established that mice and every other rodent species, unlike humans and Old-World nonhuman primates (OWPs), do not develop antibodies to α-galactopyranose (α-Gal), a highly immunogenic sugar residue expressed at the terminal nonreducing end of oligosaccharide chains from GPI-linked mucins of trypomastigotes. With the exception of OWPs, the formation of α-Gal in mammalian tissues depends on the enzymatic function of α-galactosyltransferase(s). Since rodents (unlike humans and OWPs) express α-Gal endogenously, they become immunologically tolerant to these self-structures. This is the reason why rodents are not able to produce anti- α-Gal antibodies when challenged by α-Gal bearing pathogens. In contrast, humans and OWPs naturally produce anti-α-Gal antibodies, and this immune response is usually potentiated upon infection by different pathogens, including *T. cruzi* and *Leishmania* spp. The perception that the antibody repertoire of wild type mice lacks a fundamental component of immune response (anti- α-Gal) of humans and OWP was the starting point of a productive line of immunology research^7^ ^,^ ^8^ launched by Luiz RG Travassos (EPM/UNIFESP), a pioneer in the field of parasite and fungal glycobiology. Systematic investigations carried out by Igor Almeida (at USP and University of Texas-El Paso)^9^ ^,^ ^10^ have paved the way for the design of synthetic vaccines and chemotherapy biomarkers for follow-up based on α-Gal-bearing neoglycoproteins (NGPs). With a US patent recently assigned to the University of Texas System,^11^ the PhD studies that Igor Almeida initiated in Travassos’s lab resulted in an innovative approach in human vaccination against *T. cruzi*, *Leishmania* spp. and other pathogens. Of further interest, these synthetic α-Gal-NGPs are being currently validated as biomarkers for early assessment of cure following chemotherapy with benznidazole or nifurtimox in an ongoing NIH-funded phase-2 trial (https://clinicaltrials.gov/ct2/show/NCT03981523). If successful, these molecular assays will serve as prognostic tools of therapeutic cure, hence translating to the clinical settings a biological phenomenon (lytic antibodies) originally described by Antoniana Krettli, J Romeu Cançado, and Zigman Brenner.^12^ Collectively, these classic discoveries illustrate how systematic investigations in basic Chagas disease may have broader application in translational Medicine.

As pondered by Alexander R Precioso, the prevalence of vectorial transmission of Chagas disease in Brazil is low and limited to distant geographic regions. Hence, the high cost and the complexity of logistics required for large scale clinical trials of an immunoprophylactic vaccine might be an obstacle to this pretense. Clinical studies with therapeutic vaccines (perhaps associated to chemotherapy with lower doses of benznidazole) might be safely conducted in hospital units under supervision of medical staff. However, this initiative must be preceded by preclinical studies convincingly showing that the chronic Chagas disease cardiomyopathy (CCC) is either prevented or markedly attenuated by therapeutic interventions. Another complication is that therapeutic vaccination in animals that are chronically infected with *T. cruzi* must be evaluated with parasite strains/isolates representative of various DTUs.

As mentioned earlier, there is a second reason why preclinical studies in wild-type mice may not be the ideal model for testing vaccine efficacy (prophylactic or therapeutic) against Chagas disease. For reasons that are still unclear, chronically infected mice do not consistently develop the dilated form of cardiomyopathy observed in patients. Future attempts to evaluate the cardioprotective effect of immunoprophylactic or therapeutic vaccines in chronically infected animals should be preferably performed in colonies of OWP or dogs. Considering that vaccination studies in chronically infected dogs will be operationally complex, it might be preferable to test vaccine efficacy in transgenic mice deficient of the alpha-galactosyltransferasec gene, or, alternatively, in hamsters in which this key gene will be eventually ablated. In a study published several years ago, Edécio Cunha Neto and co-workers^13^ demonstrated that a significant proportion of chronically infected hamsters developed a dilated cardiomyopathy that closely resembles human CCC. Although the hamster is known for decades as a rodent species that is highly susceptible to infection by *Leishmania* spp., immunologists comprehensibly showed low interest to investigate immunity mechanisms in hamsters because there is limited availability of tools (monoclonal antibodies) to phenotype leukocytes and tissue cells. Regarding transgenesis, so far there is only a single report, published by Chinese groups, describing CRISP/Cas-9- dependent generation of transgenic lines of hamsters. Recently, however, awareness that the Syrian hamster is highly susceptible to acute infection by Ebola and coronaviruses, including SARS-Cov-2^14^ has prompted iniciatives in R&D to supply immunoreagents for hamster research.^15^

The history of vaccination against neglected parasitic diseases is still nascent, reflecting the difficulty to cross the “valley of death” of vaccine development. While sharing his own experience as the director of WHO-TDR (1998), Carlos Morel reproduced fragments of a conversation he held in his Geneve office with Barry Bloom, an influential immunologist of the second half of the 20th century. While not minimising the hardships that immunoparasitologists faced throughout decades, we recently learned that a phase-2b randomised clinical trial to evaluate the efficacy of a modified CS-based vaccine (R21 in adjuvant Matrix-M) against malaria was concluded in Burkina Faso.^16^ The results showed 77% efficacy in a cohort study involving seasonal vaccine administration to 450 children. Although the results of phase 3 trials are still to come, this human vaccine against malaria is the outcome of a life-time project headed by Ruth and Victor Nussenzweig. Joined by several brilliant co-workers, these Brazilian-born immunoparasitologists should be credited for being the first to cross the “valley of death” in vaccine development. More importantly, millions of lives in Africa might be spared as result of their milestone discoveries.^17^

Without fear of being nostalgic, my final note goes to a conversation that I held in the early 80’s in Caxambu (Hotel Gloria) with Maurício M Rodrigues, my first scientific initiation (IC) student. One night, under the fresh evening breeze of November, over a beer, we stopped laughing about the funny scenes that Isaac Roitman created for his famous Award at the end of the Annual Meetings to discuss distant prospects of a vaccine against Chagas’ disease. With excitement on his bright eyes, the young Maurício first learned about the strategies that the NYU team was developing to produce a malarial vaccine based on CS antigen. Back to the future, after spending a post doc with Fidel Zavala at NYU, Maurício Rodriguez developed a productive career at Universidade Federal De São Paulo (UNIFESP), mostly focused on the development of an experimental vaccine against Chagas disease. Irrespective of the outcome of vaccination against Chagas disease, the mysteries behind *T. cruzi* successful adaptation in immunocompetent mammals will still defy the imagination (Figure) of multiple generations of students attending Sociedade Brasileira de Protozoologia (SBPZ) and the Annual Meetings of Basic Research on Chagas Disease.

**Figure:**
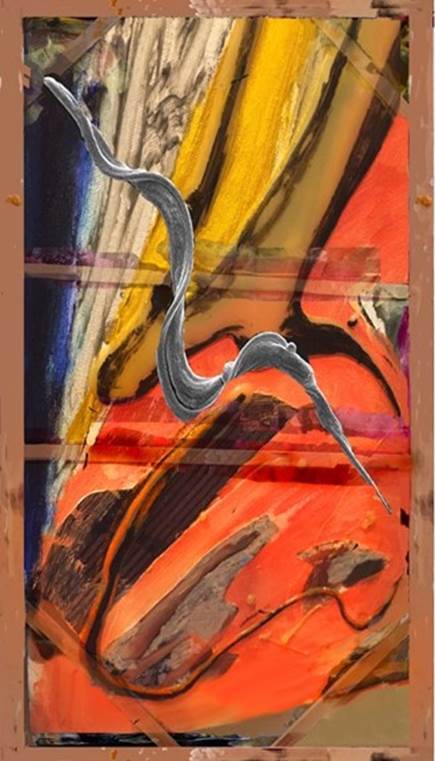
*Trypanosoma cruzi*, a flagellated protozoan seeking shelter from the inflammatory storm. Painting and digital art. Julio Scharfstein 2018. Dedicated *in memoriam* to Juliana De Meis, the Editor of this Special Issue.

Comments on the article: Camargo EP, Gazzinelli RT, Morel CM, Precioso AR. Why do we still have not a vaccine against Chagas disease? Mem Inst Oswaldo Cruz. 2021; 116: e200314.
